# *Lactobacillus plantarum* Lac16 alleviates dextran sodium sulfate-induced colitis in mice by suppressing NLRP3 inflammasome overactivation through microbiota-derived isobutyric acid

**DOI:** 10.1128/mbio.02392-25

**Published:** 2025-10-31

**Authors:** Yuanhao Zhou, Baikui Wang, Qi Wang, Fei Wang, Xiaozhou Mou, Li Gong, Weifen Li

**Affiliations:** 1 Department of Rehabilitation Medicine, Center for Rehabilitation Medicine, Rehabilitation and Sports Medicine Research Institute of Zhejiang Province, Zhejiang Provincial People’s Hospital (Affiliated People’s Hospital), Hangzhou Medical College 117839, Hangzhou, Zhejiang, China; 2 Zhejiang Key Laboratory of Tumor Molecular Diagnosis and Individualized Medicine, Clinical Research Institute, Zhejiang Provincial People’s Hospital, Affiliated People’s Hospital, Hangzhou Medical College 117839, Hangzhou, Zhejiang, China; 3 Key Laboratory of Molecular Animal Nutrition of the Ministry of Education, Institute of Animal Nutrition and Feed Sciences, College of Animal Sciences, Zhejiang University 366125, Hangzhou, China; 4 Key Laboratory of Animal Nutrition and Feed Science (Eastern of China) of the Ministry of Agriculture, Institute of Animal Nutrition and Feed Sciences, College of Animal Sciences, Zhejiang University, Hangzhou, China; 5 Key Laboratory of Animal Feed and Nutrition of Zhejiang Province, Institute of Animal Nutrition and Feed Sciences, College of Animal Sciences, Zhejiang University, Hangzhou, China; 6 MOE Key Laboratory of Macromolecular Synthesis and Functionalization, Department of Polymer Science and Engineering, Zhejiang University 12377, Hangzhou, China; 7 Key Laboratory of Systems Health Science of Zhejiang Province, School of Life Science, Hangzhou Institute for Advanced Study, University of Chinese Academy of Sciences, Hangzhou, China; 8 Guangdong Provincial Key Laboratory of Animal Molecular Design and Precise Breeding, School of Life Science and Engineering, Foshan University 118208 , Foshan, China; College of Veterinary Medicine, Cornell University, Ithaca, New York, USA

**Keywords:** colitis, *Lactobacillus plantarum* Lac16, NLRP3 inflammasome, microbiota, isobutyric acid

## Abstract

Inflammatory bowel disease (IBD) is a chronic intestinal inflammatory disorder characterized by immune dysregulation. Therapeutic strategies targeting gut microbiota modulation have been proven to be effective against IBD. This study sought to explore the protective effects of *Lactobacillus plantarum* Lac16 against dextran sulfate sodium (DSS)-induced colitis and to elucidate its underlying mechanisms. Results illustrated that Lac16 administration significantly ameliorated colitis symptoms, as evidenced by reduced weight loss, attenuated colon shortening, and decreased disease activity index scores. Furthermore, Lac16 treatment restored intestinal barrier integrity, modulated inflammatory cytokine levels, promoted macrophage polarization toward an anti-inflammatory phenotype, and suppressed NOD-like receptor protein 3 (NLRP3) inflammasome overactivation in the colon. Lac16 treatment effectively remodeled gut microbiota composition by significantly increasing the abundance of beneficial bacteria, particularly *Alloprevotella* and *Dubosiella*, while reducing the abundance of potentially pathogenic bacteria such as *Bacteroides* and *Helicobacter*. It also elevated acetic acid and isobutyric acid levels among microbiota-derived short-chain fatty acids. The pseudo-germ-free mouse model confirmed that Lac16 inhibits NLRP3 inflammasome overactivation and ameliorates colitis symptoms by modulating the gut microbiota. Moreover, the fecal microbiota transplantation (FMT) mouse model demonstrated that these protective benefits are transferable through FMT. Subsequently, isobutyric acid was found to suppress NLRP3 inflammasome overactivation and ameliorate colitis *in vivo* while also attenuating inflammatory injury *in vitro*. Collectively, these findings illustrate that Lac16 alleviates colitis and provides gastrointestinal protection by suppressing NLRP3 inflammasome overactivation through a gut microbiota-dependent mechanism, with microbiota-derived isobutyric acid identified as the pivotal mediator in this protective process.

**IMPORTANCE:**

This study establishes that *Lactobacillus plantarum* Lac16 alleviates DSS-induced colitis through gut microbiota-dependent mechanisms. Lac16 administration significantly ameliorated colitis symptoms while restoring intestinal barrier integrity, promoting anti-inflammatory macrophage polarization, and suppressing NLRP3 inflammasome overactivation. The pseudo-germ-free mouse model provided definitive evidence that Lac16’s suppression of NLRP3 inflammasome overactivation requires gut microbiota. Fecal microbiota transplantation verified the causal role of microbiota in mediating Lac16’s therapeutic benefits. Notably, Lac16 reshaped microbial composition, elevating beneficial genera (*Alloprevotella* and *Dubosiella*) while suppressing pathogenic genera (*Bacteroides* and *Helicobacter*). Crucially, Lac16 increased microbiota-derived short-chain fatty acids, particularly isobutyric acid. Both *in vivo* and *in vitro* experiments confirmed that isobutyric acid significantly contributes to anticolitic effects and suppresses NLRP3 activation. These findings elucidate a novel mechanism by which Lac16 ameliorates colitis via (i) microbiota-dependent NLRP3 inflammasome modulation and (ii) isobutyric acid-mediated protective effects. This work provides important insights into probiotic mechanisms and supports targeting microbial metabolic networks for IBD intervention.

## INTRODUCTION

Inflammatory bowel disease (IBD), encompassing Crohn’s disease and ulcerative colitis (UC), is a chronic and recurrent intestinal inflammatory disorder of the intestine characterized by abdominal pain, diarrhea, hematochezia, and maldigestion (1, 2). The etiology of IBD remains ambiguous. Nevertheless, research indicates that impaired mucosal barrier function, genetic predisposition, environmental triggers, dysregulated immune responses, and gut microbiota dysbiosis collectively contribute to the occurrence and progression of IBD (3–5).

Pyroptosis is a caspase-1-dependent programmed cell death mediated by canonical inflammasomes, particularly the NOD-like receptor protein 3 (NLRP3) inflammasome. This inflammatory cell death pathway plays a crucial role in regulating immune responses through cellular lysis and pro-inflammatory cytokine release (6, 7). As a prototypical inflammasome, the NLRP3 inflammasome detects diverse inflammatory stimuli and facilitates IL-1β/IL-18 maturation, whose dysregulated overactivation is critically implicated in IBD pathogenesis (8, 9). Excessive activation of the NLRP3 inflammasome triggers damage to intestinal epithelial cells and elevates intestinal permeability, thereby facilitating the translocation of luminal bacteria and toxins across the mucosal barrier into the lamina propria, ultimately exacerbating immune responses (10). Given the pivotal role of the NLRP3 inflammasome in the pathogenesis of IBD, this multiprotein complex has emerged as a promising therapeutic target (11).

In the intestinal tract, bacteria, fungi, archaea, viruses, and other microorganisms form an intricately interconnected ecological community known as the gut microbiota (12). The gut microbiota and its metabolic products, including but not limited to short-chain fatty acids (SCFAs), bile acids, aromatic amino acids, and others, interact with intestinal epithelial cells to preserve mucosal barrier function while simultaneously regulating immune responses through bidirectional communication with the host immune system (13–15). Microbial dysbiosis, characterized by an imbalanced gut microbiota composition, impairs intestinal mucosal barrier integrity and triggers inflammatory responses (16). The existence of microbial dysbiosis in IBD patients provides compelling evidence for a pathophysiological association between gut microbiota disruption and IBD development, highlighting the microbiota’s fundamental role in intestinal homeostasis (17). Current therapeutic strategies for IBD targeting gut microbiota modulation have attracted substantial research and clinical attention (18).

Probiotics are defined as “live microorganisms that, when administered in adequate amounts, confer a health benefit on the host” (19). Accumulating evidence demonstrates that probiotic administration can alleviate colitis symptoms, modulate immune responses, and maintain gut microbiota homeostasis (20–22). Our previous study identified that *Lactobacillus plantarum* Lac16, a strain isolated by our laboratory, significantly improves intestinal histomorphology, modulates gut microbiota composition, and attenuates infection-induced inflammatory responses (23–25). Nevertheless, the potential of Lac16 to alleviate colitis symptoms and modulate the associated immune-microbial dysregulation remains to be fully explored.

In this study, we investigated the protective mechanisms of the probiotic Lac16 against dextran sulfate sodium (DSS)-induced colitis. Our findings demonstrate that Lac16 ameliorates intestinal inflammation by preserving intestinal barrier integrity, modulating immune responses, suppressing excessive NLRP3 inflammasome activation, and maintaining gut microbiota homeostasis. Furthermore, we identified microbiota-derived isobutyric (IB) acid as the key mediator underlying these protective effects.

## MATERIALS AND METHODS

### Bacterial strains

The probiotic strain Lac16 (deposited at the China Center for Type Culture Collection No. M2016259) was cultured in Mann-Rogosa-Sharpe broth at 37°C for 18 h, harvested by centrifugation, resuspended in sterile phosphate-buffered saline (PBS, pH = 7.4), and stored at 4℃ for experimental use.

### Animal models

Male C57BL/6 mice (4 weeks old) were obtained from SLAC Laboratory Animal Co., Ltd. (Shanghai, China), and maintained under specific pathogen-free conditions with *ad libitum* access to food and water throughout the acclimatization period.

#### Experiment I

Mice were randomly divided into three experimental groups (*n* = 10 per group): control, DSS, and Lac16 + DSS. The Lac16 + DSS group received 2 × 10⁸ CFU Lac16 in 200 µL PBS via daily oral gavage, while the control and DSS groups received an equal volume of sterile PBS. On day 21, colitis was induced in the DSS and Lac16 + DSS groups by administering 2.5% (wt/vol) DSS (MP Biomedicals, Santa Ana, CA, USA) in drinking water for 7 days.

#### Experiment II

Mice were randomly divided into two experimental groups (*n* = 5 per group): Abx + Lac16 + DSS and Lac16 + DSS. The Abx + Lac16 + DSS group received a 7-day pretreatment with an antibiotic cocktail (colistin 850 U/mL, gentamicin 0.5 mg/mL, ampicillin 1 mg/mL, vancomycin 0.2 mg/mL, and fluconazole 0.5 mg/mL; Sigma-Aldrich, St. Louis, MO, USA) in drinking water. On day 7, mice in both groups received 2 × 10⁸ CFU of Lac16 via daily oral gavage for 14 consecutive days. Colitis was induced on day 14.

#### Experiment III

Donor mice were randomly divided into four groups (*n* = 5 per group): control, DSS, Lac16, and Lac16 + DSS. Donor mice in the Lac16 group received 2 × 10⁸ CFU of Lac16 via daily oral gavage, while other groups were treated identically to experiment I. On days 26–28, fresh feces were collected, weighed, and homogenized in sterile PBS (100 mg feces /mL). After centrifugation at 200 × *g* for 5 min, the supernatant was collected and filtered through a 70 µm filter to remove impurities.

Recipient mice were randomly divided into four groups (*n* = 5 per group): fecal microbiota transplantation (FMT)-control, FMT-DSS, FMT-Lac16, and FMT-Lac16 + DSS. Throughout the experiment, recipient mice received 200 µL of fecal suspension from their respective donor groups via daily oral gavage. Colitis induction was initiated on day 14.

#### Experiment IV

Mice were randomly divided into three experimental groups (*n* = 5 per group): control, DSS, and IB acid + DSS. Mice in the IB acid + DSS group received a 3 week pretreatment with 150 mM isobutyrate (Macklin, Shanghai, China) in drinking water. On day 21, colitis was induced in both the DSS and the IB acid + DSS groups.

### Disease activity index

During the final 7 days of the experiment, mice were weighed and scored daily for pathological features as previously described (26). Briefly, the disease activity index (DAI) for evaluating colitis severity was calculated as the sum of scores for weight loss (0, none; 1, 1%–5%; 2, 5%–10%; 3, 10%–15%; and 4, >15%), stool consistency (0, normal; 2, loose; and 4, diarrhea), and hematochezia (0, normal; 2, slight bleeding; and 4, gross bleeding), with all parameters assessed daily in each mouse.

### Histopathology

Colon tissue was fixed in 4% (vol/vol) paraformaldehyde (Beyotime, Shanghai, China) and dehydrated, followed by paraffin embedding, sectioning, and staining with hematoxylin and eosin or alcian blue-periodic acid-Schiff. Images were acquired via microscope (Olympus, Tokyo, Japan). Histological scoring was performed according to the established scoring system described by Stillie and Stadnyk (27).

### Transmission electron microscopy

Colon tissue was fixed overnight at 4°C in 2.5% (vol/vol) glutaraldehyde (Sinopharm, Shanghai, China). After that, the tissues were sequentially fixed, dehydrated, embedded, sectioned, stained, and observed using a transmission electron microscopy (Hitachi H-7650; Hitachi, Ibaraki, Japan).

### Immunohistochemistry

Colon sections were successively dewaxed, rehydrated, antigen repaired, blocked, incubated with primary antibodies (mucin 2, NLRP3, and myeloperoxidase [MPO]; Servicebio, Wuhan, China) and secondary antibody (Servicebio), stained, counterstained, dehydrated, clarified, and mounted for imaging using a microscope (Olympus).

### Immunofluorescence

Colon sections were successively dewaxed, rehydrated, antigen repaired, blocked, and incubated with primary antibodies (occludin + zona occludens-1 [zo-1], F4/80 + inducible nitric oxide synthase [iNOS]/arginase 1 [Arg-1]; Servicebio) and secondary antibodies (Servicebio). Nuclei were counterstained with 4′,6-diamidino-2-phenylindole (Beyotime), and samples were imaged using laser scanning confocal microscopy (Zeiss, Oberkochen, Germany) with subsequent quantification performed using ImageJ software.

### Hematological analysis

Hematological parameters were analyzed using an automated hematology analyzer (Yuyanbio, Shanghai, China).

### Biochemical analysis

MPO activity and cytokine levels (IL-1β, IL-6, TNF-α, IL-18, IL-10, and IFN-γ) in the colon were determined using an assay kit. Serum lipopolysaccharide (LPS) concentrations were quantified by enzyme-linked immunosorbent assay (Nanjing Jiancheng Biology Engineering Institute, Nanjing, China).

### Quantitative real-time PCR

Total RNA was extracted from colon tissues using RNAiso Plus (Takara, Dalian, China), followed by reverse transcription. Quantitative real-time PCR was performed on a StepOne Plus system (Applied Biosystems, Carlsbad, CA, USA) with β-actin as the endogenous control. Primer sequences are listed in Table S1. Relative gene expressions were quantified using the 2^−∆∆Ct^ method (28).

### Western blot analysis

Colon samples were homogenized in lysis buffer, and protein concentrations were determined using a BCA assay kit (Beyotime). Proteins were separated by SDS-PAGE and transferred to polyvinylidene fluoride membranes (Millipore, Billerica, MA, USA). After blocking, membranes were incubated overnight at 4°C with the following primary antibodies: NLRP3 (HuaBio, Hangzhou, China), apoptosis-associated speck-like protein containing a C-terminal CARD (ASC; Abclonal, Wuhan, China), caspase-1 (AdipoGen, Seoul, Korea), IL-1β (Sigma-Aldrich), and β-actin (Abcam, Cambridge, UK). Membranes were incubated with horseradish peroxidase (HRP)-conjugated secondary antibodies (HuaBio), followed by chemiluminescent detection using an HRP substrate kit (Millipore). Ultimately, protein bands were detected using imaging equipment (Tanon, Shanghai, China) and quantified using ImageJ software.

### Intestinal microbiota analysis

The V3–V4 hypervariable regions of bacterial 16S rRNA genes were amplified from cecal content DNA using primers 338F (5′- ACTCCTACGGGAGGCAGCA-3′) and 806R (5′-GGACTACHVGGGTWTCTAAT-3′). The PCR amplicons were sequenced on the Illumina MiSeq platform. Raw sequences were quality-filtered and clustered into operational taxonomic units at 97% similarity using QIIME software. Microbial α-diversity (Ace, Chao1, Shannon, and Simpson indices) and β-diversity were analyzed using the QIIME software. β-Diversity was visualized through principal coordinate analysis (PCoA) plots. The relative abundance of microbiota was analyzed across several taxonomic levels.

### SCFA quantitative analysis

The method for determining SCFA concentrations was adapted from Gong et al. (29) with modifications. Fecal samples were homogenized and centrifuged. The supernatant was mixed with 25% (wt/vol) metaphosphoric acid, incubated at −20°C for 24 h, then centrifuged and filtered. SCFAs were detected using the NEXIS GC-2030 ATF (Shimadzu Group Company, Kyoto, Japan).

### Determination of the elimination effect of intestinal microbiota

Fecal samples were collected aseptically, weighed, homogenized, serially diluted, and plated on brain heart infusion agar. Colony growth was documented photographically after 48 h of incubation.

### Cell culture

HT-29 cells were cultured in Dulbecco’s modified Eagle’s F12 ham medium (DMEM/F12) augmented with 10% fetal bovine serum, 100 U/mL penicillin, and 100 µg/mL streptomycin. Cells were maintained at an atmosphere of 37°C and 5% CO_2_.

Bone marrow-derived macrophages (BMDMs) were isolated from femurs and tibias of mice. Both ends of the bones were cut, and the bone marrow was flushed with sterile PBS. The cell suspension was collected, and red blood cells were lysed. After centrifugation, the cells were resuspended in complete DMEM supplemented with 10 ng/mL macrophage colony-stimulating factor (M-CSF). After 72 h of culture, fresh complete DMEM with 10 ng/mL M-CSF was added for another 72 h. On day 7, cells were trypsinized, seeded, and cultured in M-CSF-free complete DMEM.

### Cytotoxicity assay

Cytotoxicity assay was evaluated using a CCK-8 assay (Beyotime). When HT-29 cells reached 80% confluence, they were treated with isobutyric acid (0.1, 1.0, 10.0, and 100.0 µM and 1.0 and 10.0 mM) for 24 h. Subsequently, cells were incubated in serum-free DMEM/F12 containing 10% (vol/vol) CCK-8 solution, and absorbance was measured at 450 nm. Cell viability was assessed according to the manufacturer’s protocol.

To evaluate the protective effect of isobutyric acid, HT-29 cells were co-treated with increasing concentrations of isobutyric acid (0.001, 0.01, 0.1, 1.0, 10.0, and 100 µM and 1 mM) and 10 µg/mL LPS (Sigma-Aldrich) for 24 h. Cell viability was then determined as described previously.

To assess the anti-inflammatory effects of isobutyric acid, BMDMs were co-treated with 1 mM isobutyric acid and 1 µg/mL LPS for 24 h, followed by cell viability analysis as previously described.

### Apoptosis assay

Cell apoptosis was analyzed using an Annexin V-FITC/PI Apoptosis Detection Kit (Beyotime). HT-29 cells treated with 1 mM isobutyric acid and 10 µg/mL LPS for 24 h, then harvested, washed, stained, and examined using flow cytometry (Becton Dickinson, Parsippany, NJ, USA).

### Scratch wound healing assay

Upon reaching 90% confluence, a wound was scratched in HT-29 cells, followed by PBS washing to remove debris. Cells were then treated with 1 mM isobutyric acid and 10 µg/mL LPS for 24 h. Wound healing progression was quantified by measuring scratch width at 0 and 24 h using an EVOS M7000 Imaging System (Thermo Fisher Scientific, Waltham, MA, USA).

### Statistical analysis

Statistical analyses were performed using SPSS software. Data are presented as mean ± SD. Between-group comparisons were analyzed by two-tailed Student’s *t*-test, while multigroup comparisons used one-way analysis of variance with Tukey’s post hoc test. Differences were deemed statistically significant when *P* < 0.05. Graphical representations were created using GraphPad Prism 9 (San Diego, CA, USA).

## RESULTS

### Lac16 pretreatment mitigated DSS-induced colitis

**Fig 1:**
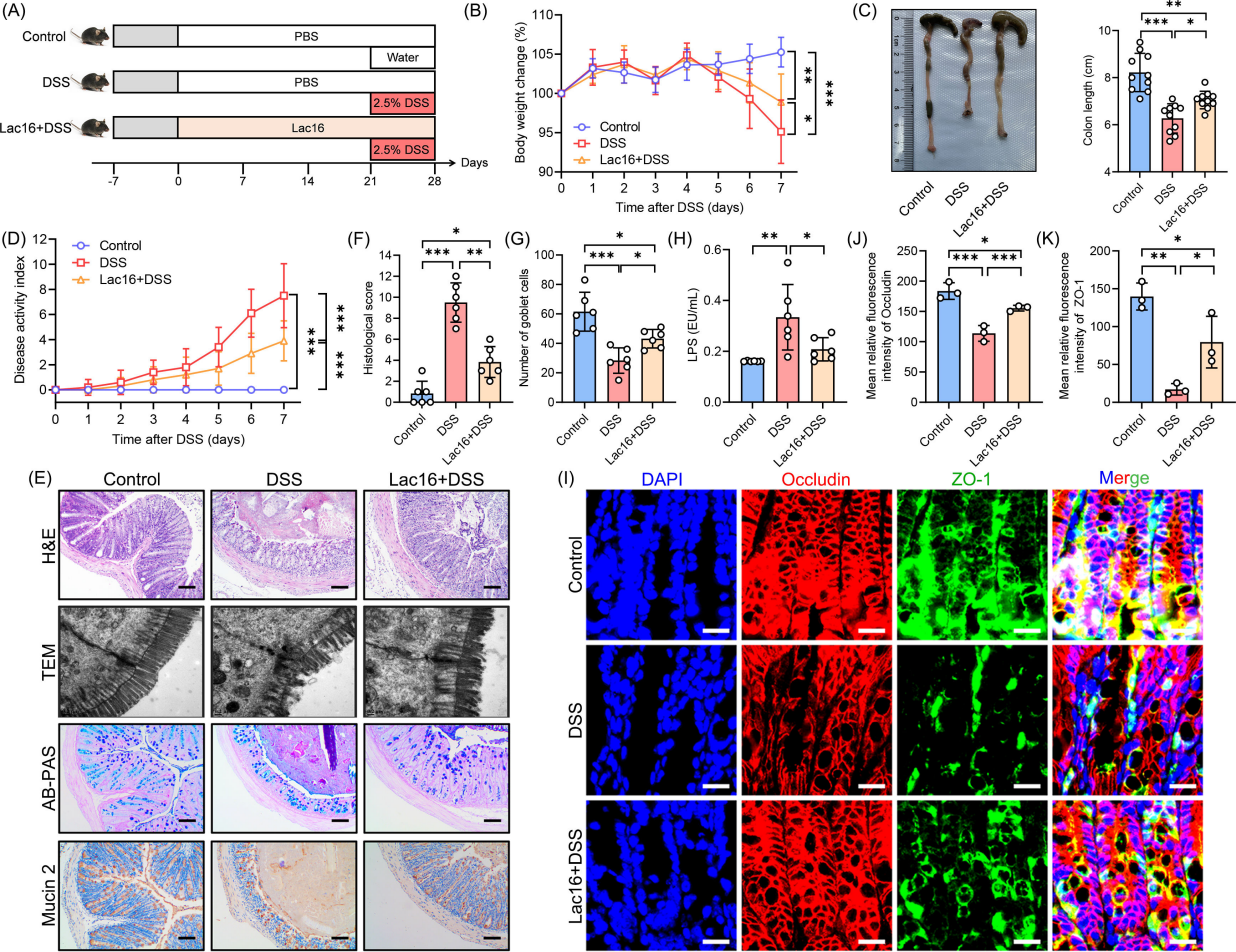
Lac16 pretreatment alleviated colitis. (**A**) Experimental design of experiment I. (**B**) Body weight change. (**C**) Representative pictures of colons and the lengths of the colons. (**D**) DAI assessment. (B–D) *n* = 10 per group. (**E**) Representative micrographs of hematoxylin and eosin (H&E) (scale bar, 100 µm), transmission electron microscopy (TEM) (scale bar, 0.2 µm), alcian blue-periodic acid-Schiff (AB-PAS) (scale bar, 100 µm), and mucin 2 (scale bar, 100 µm). (**F**) Histological score of H&E staining. (**G**) Goblet cell counting in AB-PAS staining. (**H**) Contents of LPS in the serum. (**I**) Immunofluorescence analysis for occludin (red) and zo-1 (green) in the colon. Nuclei (4′,6-diamidino-2-phenylindole [DAPI], blue) (scale bar, 20 µm). (**J and K**) Mean relative fluorescence intensity of occludin and zo-1 in panel I. (**E–K**) *n* = 3 or 6. Means ± SD; *P < 0.05, **P < 0.01, ***P < 0.001. Significance was determined by one-way analysis of variance with Tukey’s post hoc test.

To investigate the effects of Lac16 on DSS-induced colitis, mice were administered 2 × 10^8^ CFU of Lac16 for 4 weeks, with colitis induction occurring during the final 7 days (Fig. 1A). DSS administration induced significant weight loss, colon shortening, and elevated DAI scores in mice, all of which were markedly attenuated by Lac16 (Fig. 1B through D). Pretreatment with Lac16 significantly ameliorated multiple DSS-induced colonic pathologies, including ulcer formation, inflammatory infiltration, crypt loss, microvillus damage, goblet cell depletion, and downregulation of mucin 2 expression (Fig. 1E through G; Fig. S1A). Furthermore, Lac16 pretreatment significantly decreased serum LPS levels (Fig. 1H), a well-established biomarker of intestinal barrier dysfunction (30). Consistent with the observed barrier dysfunction, DSS challenge markedly reduced colonic levels of key tight junction proteins (occludin, zo-1, and claudin-1), while Lac16 pretreatment largely preserved their expression (Fig. 1I through K; Fig. S1B through D).

### Lac16 pretreatment attenuated DSS-induced inflammation

**Fig 2:**
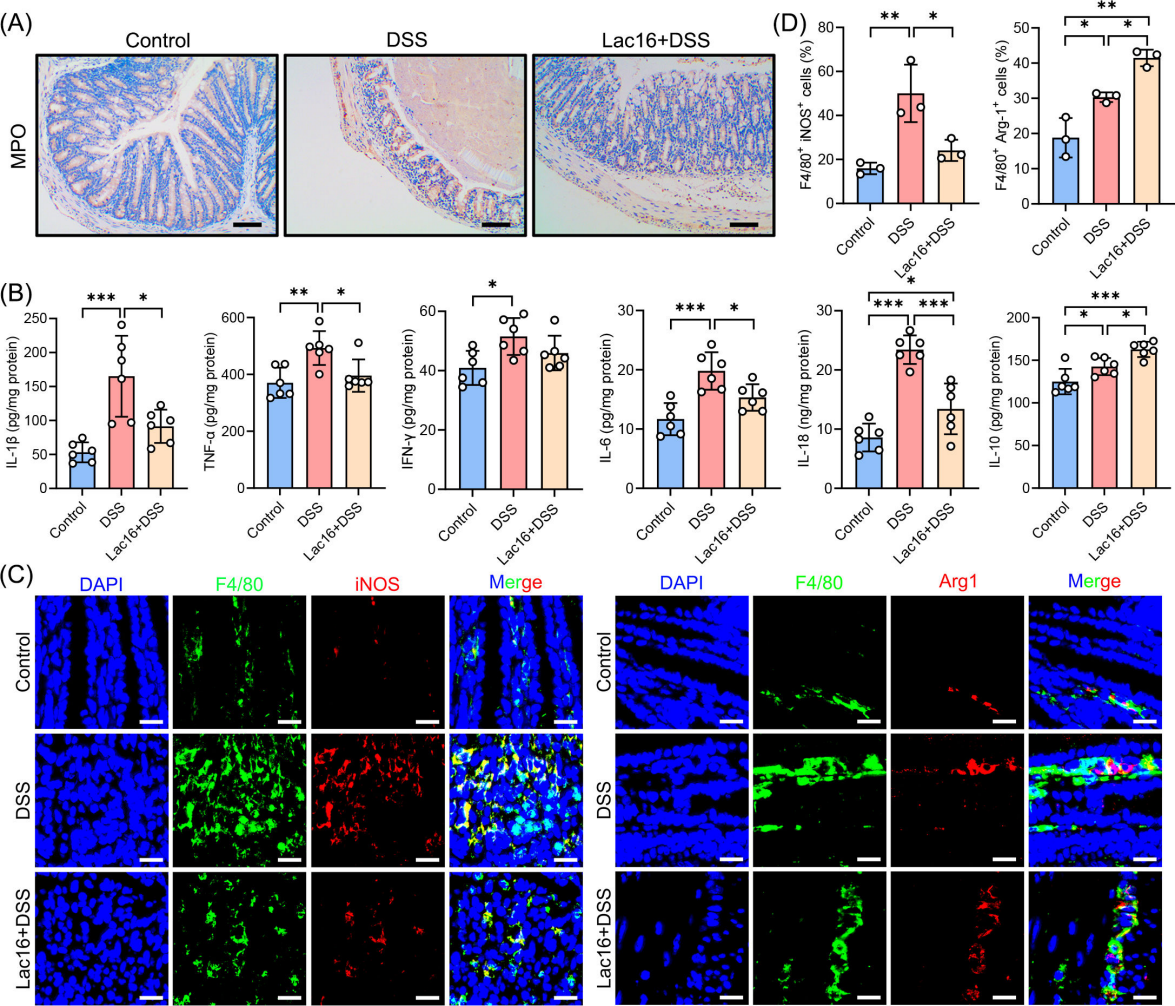
Lac16 pretreatment attenuated DSS-induced inflammation. (**A**) Representative micrographs of MPO (scale bar, 100 µm). (**B**) Contents of the IL-1β, TNF-α, IFN-γ, IL-6, IL-18, and IL-10 in the colonic tissue. (**C**) Immunofluorescence analysis for F4/80^+^ (green) iNOS^+^ (red) cells or F4/80^+^ (green) Arg1^+^ (red) cells in the colon. Nuclei (DAPI, blue) (scale bar, 20 µm). (**D**) The proportion of F4/80^+^ iNOS^+^ cells or F4/80^+^ Arg-1^+^ cells in panel C. (**A–D**) *n* = 3 or 6. Means ± SD; *P < 0.05, **P < 0.01, ***P < 0.001. Significance was determined by one-way analysis of variance with Tukey’s post hoc test.

To assess the impact of Lac16 on systemic and colonic inflammation, we evaluated hematological parameters, MPO activity (as a marker of neutrophil infiltration), inflammatory cytokine levels, and intestinal macrophage polarization. Notably, the DSS challenge significantly increased circulating leukocyte populations (white blood cells, monocytes, granulocytes, and lymphocytes), effects that were substantially attenuated by Lac16 pretreatment (Fig. S2A). Furthermore, DSS treatment significantly elevated both colonic MPO expression and activity, indicating severe inflammation, while Lac16 intervention markedly attenuated these effects (Fig. 2A; Fig. S2B). Consistently, Lac16 significantly decreased colonic levels of pro-inflammatory cytokines (IL-1β, TNF-α, IL-6, and IL-18) while concurrently elevating the anti-inflammatory cytokine IL-10 (Fig. 2B). DSS treatment significantly increased the proportion of pro-inflammatory M1 macrophages (F4/80^+^iNOS^+^) while also elevating anti-inflammatory M2 macrophages (F4/80^+^Arg-1^+^), with Lac16 intervention both attenuating the M1 response and further augmenting the M2 population (Fig. 2C and D). Both M1-associated genes (iNOS, IL-1β, TNF-α, and IL-12/p40) and M2-associated markers (Arg1, MR, Ym1, and Fizz1) showed concordant expression patterns at the transcriptional level, mirroring the protein-level observations of macrophage polarization (Fig. S2C).

### Lac16 pretreatment relieved the activation of colonic NLRP3 inflammasome caused by DSS treatment

**Fig 3:**
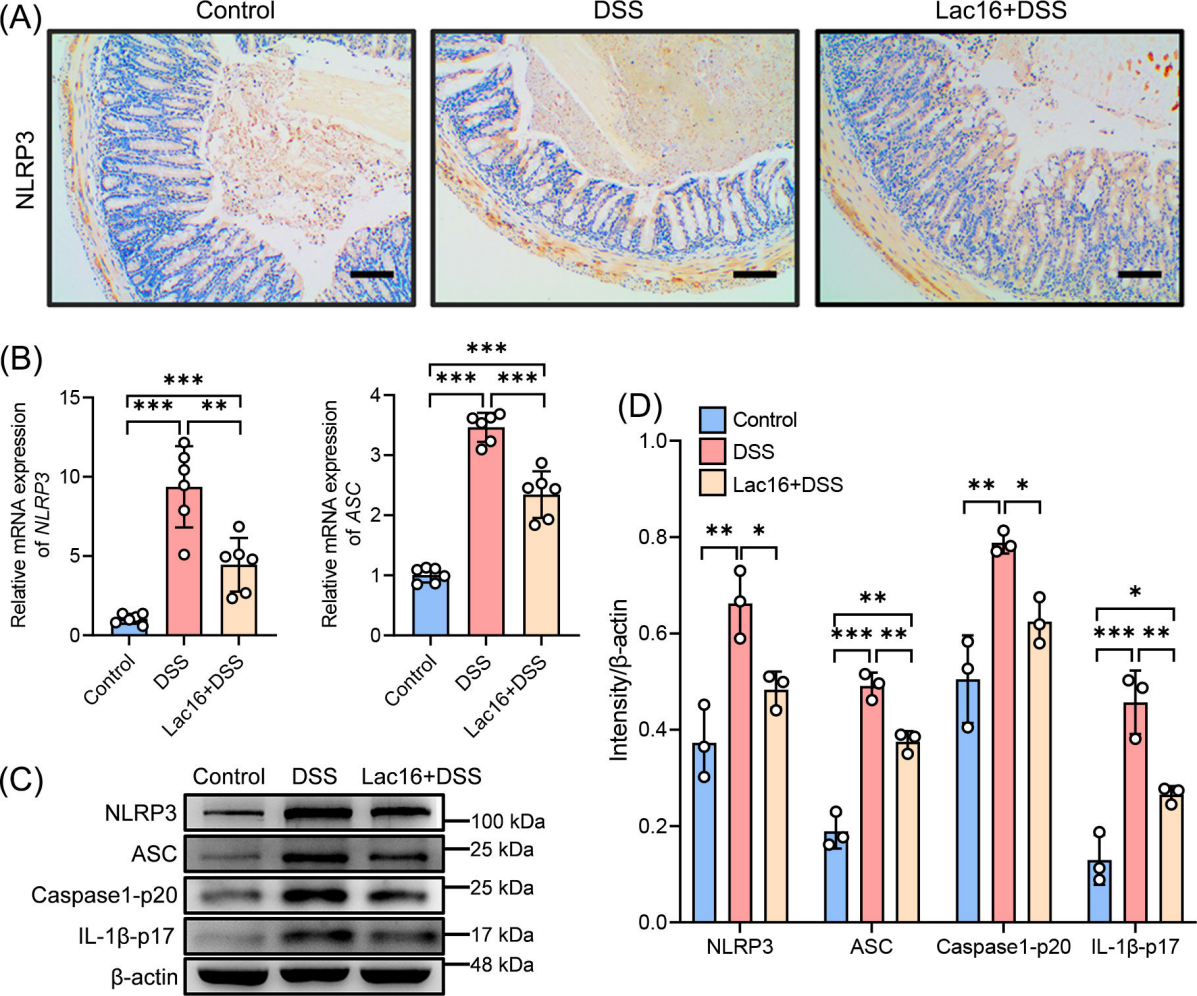
Lac16 alleviated the activation of colonic NLRP3 inflammasome caused by colitis. (**A**) Representative micrographs of NLRP3 (scale bar, 100 µm). (**B**) Relative gene expression of NLRP3 and ASC. (**C**) Western blot of the NLRP3 inflammasome pathway. (**D**) Quantitative analysis of panel C. (**A–D**) *n* = 3 or 6. Means ± SD; *P < 0.05, **P < 0.01, ***P < 0.001. Significance was determined by one-way analysis of variance with Tukey’s post hoc test.

Consistent with established links between macrophage polarization and NLRP3 inflammasome activation (7), our analysis demonstrated that DSS challenge significantly upregulated both NLRP3 protein expression and gene transcription in colonic tissue, effects that were markedly attenuated by probiotic pretreatment (Fig. 3A and B). Concurrently, the same trend was obtained in ASC, another crucial protein in the NLRP3 inflammasome pathway (Fig. 3B). Western blot analysis demonstrated significant upregulation of NLRP3 inflammasome pathway components in DSS-treated mice, including NLRP3, ASC, cleaved caspase-1 (p20), and mature IL-1β (p17), indicating robust inflammasome activation (Fig. 3C and D). Notably, Lac16 pretreatment substantially attenuated the expression of all these proteins.

### Intestinal microbiota is necessary for Lac16 to relieve colitis

**Fig 4:**
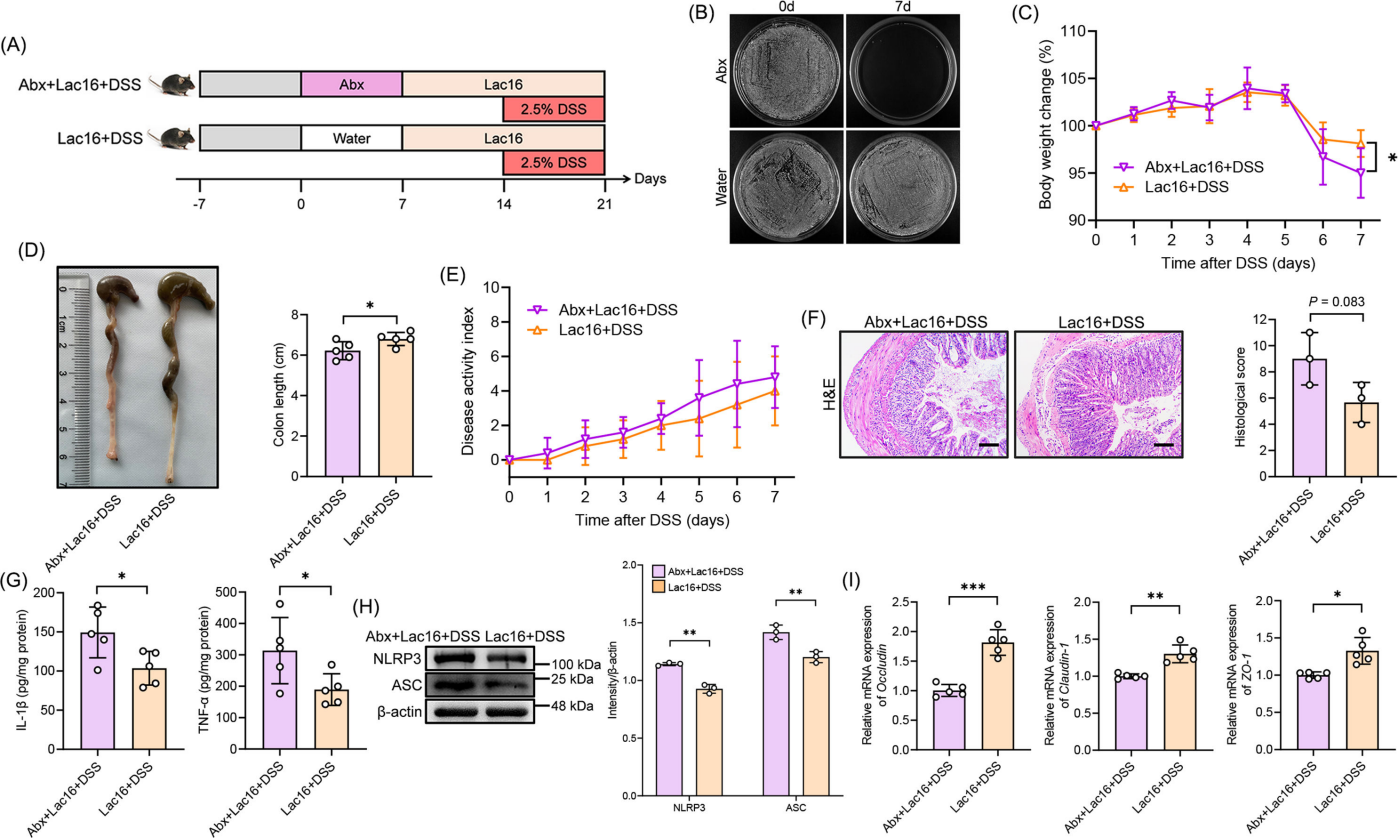
The intestinal microbiota was crucial for Lac16 to prevent colitis. (**A**) Experimental design of experiment II. (**B**) The elimination effect of Abx on intestinal microbiota. (**C**) Body weight change. (**D**) Representative pictures of colons and the lengths of the colons. (**E**) DAI assessment. (**F**) Representative micrographs of H&E (scale bar, 100 µm) and histological score. (**G**) IL-1β and TNF-α contents in the colon. (**H**) Western blot of the NLRP3 pathway and the quantitative analysis. (**I**) Relative gene levels of occludin, claudin-1, and zo-1. (**B–I**) *n* = 3 or 5. Means ± SD; *P < 0.05, **P < 0.01, ***P < 0.001. Significance was determined by two-tailed Student’s *t*-test.

To investigate the role of gut microbiota in Lac16-mediated amelioration of colitis, mice received Abx pretreatment via drinking water to deplete intestinal microbiota prior to Lac16 administration and DSS challenge (Fig. 4A). After 1 week of Abx administration, fecal microorganisms in the Abx + Lac16 + DSS group showed a dramatic reduction, indicating effective gut microbiota elimination (Fig. 4B). Following microbiota depletion, the Abx + Lac16 + DSS group exhibited significant reductions in body weight and colon length, while the DAI score remained unchanged (Fig. 4C through E). In the absence of gut microbiota, the protective effect of Lac16 pretreatment against DSS-induced colonic epithelial damage was partially attenuated (Fig. 4F). Concurrently, levels of IL-1β and TNF-α, as well as NLRP3 and ASC protein expression, were significantly elevated compared to the Lac16 + DSS group (Fig. 4G and H). Conversely, the gene expression of occludin, claudin-1, and zo-1 of the Abx + Lac16 + DSS group was significantly reduced compared to the Lac16 + DSS group (Fig. 4I). These phenomena suggested that Lac16 had reduced relieving effects on colitis after the removal of the gut microbiota.

### FMT confirmed the important role of intestinal microbiota in the anticolitis benefits of Lac16

**Fig 5:**
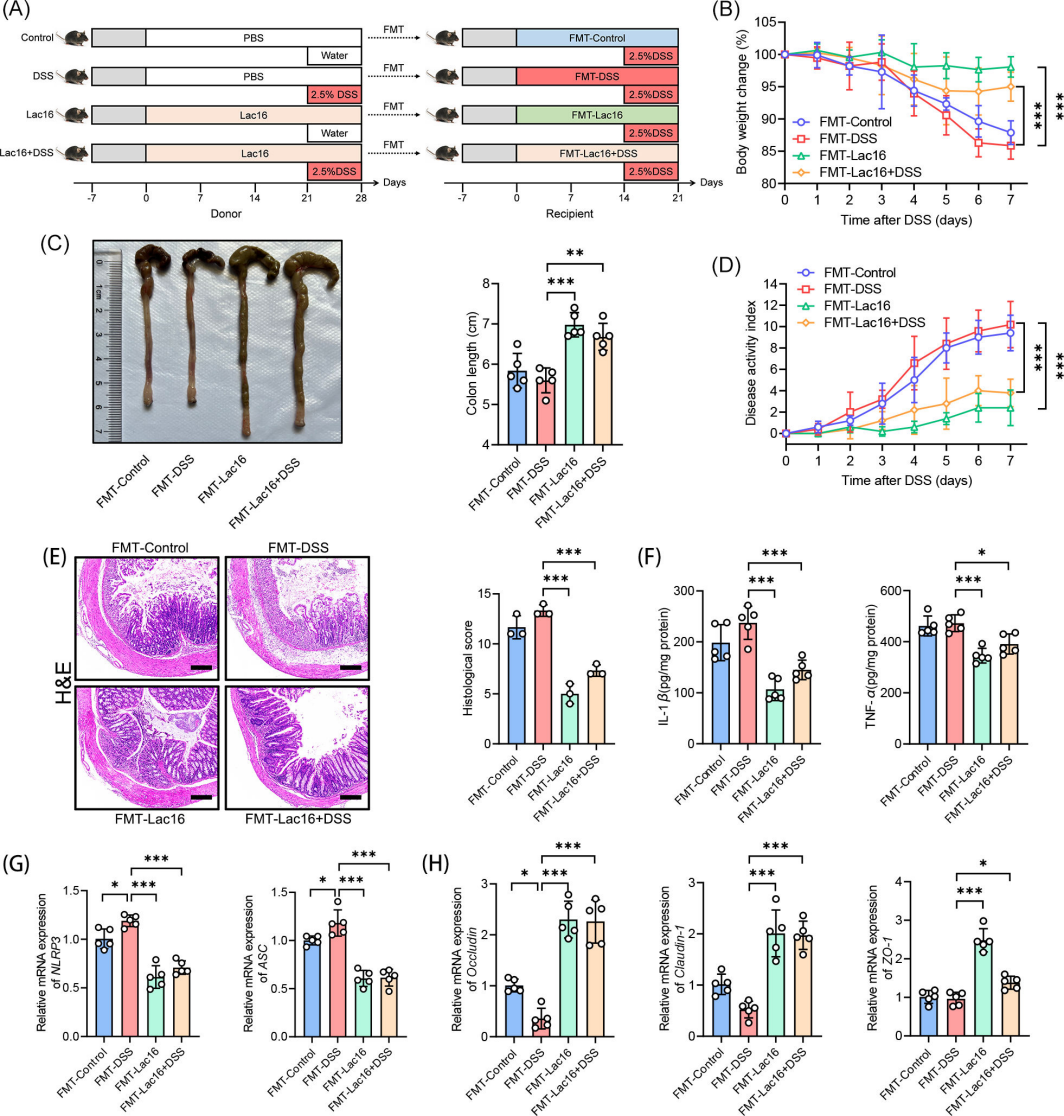
FMT from Lac16-treated mice mitigated colitis. (**A**) Experimental design of experiment III. (**B**) Body weight change. (**C**) Representative pictures of colons and the lengths of the colons. (**D**) DAI assessment. (**E**) Representative micrographs of H&E (scale bar, 100 µm) and histological score. (**F**) Concentrations of the IL-1β and TNF-α in the colon. (**G**) Relative gene levels of NLRP3 and ASC. (**H**) Relative gene levels of occludin, claudin-1, and zo-1. (**B to H**) *n* = 3 or 5. Means ± SD; *P < 0.05, **P < 0.01, ***P < 0.001. Significance was determined by one-way analysis of variance with Tukey’s post hoc test.

To investigate the role of Lac16-modulated microbiota in colitis amelioration, we performed FMT from donor mice (with or without Lac16 treatment) into recipient mice with DSS-induced colitis (Fig. 5A). FMT experiments revealed that recipient mice receiving microbiota from Lac16-treated donors (Lac16-FMT and Lac16 + DSS FMT groups) showed significantly attenuated colitis symptoms compared to DSS-FMT controls (Fig. 5B through D). The FMT-Lac16 and FMT-Lac16 + DSS groups exhibited significantly attenuated colonic epithelial damage, accompanied by reduced levels of the pro-inflammatory cytokines IL-1β and TNF-α, as well as downregulated gene expression of the inflammasome components NLRP3 and ASC (Fig. 5E through G). The colonic gene expression of occludin, claudin-1, and zo-1 was significantly elevated in recipients of microbiota from Lac16-treated or Lac16 + DSS-treated donors compared to those receiving microbiota from DSS-treated donors (Fig. 5H). Collectively, these findings demonstrate that FMT from Lac16-treated donors recapitulates the probiotic’s protective effects against colitis, further confirming the crucial contribution of microbiota in the anticolitis benefits of Lac16.

### Lac16 pretreatment ameliorated DSS-induced microbial dysbiosis

**Fig 6:**
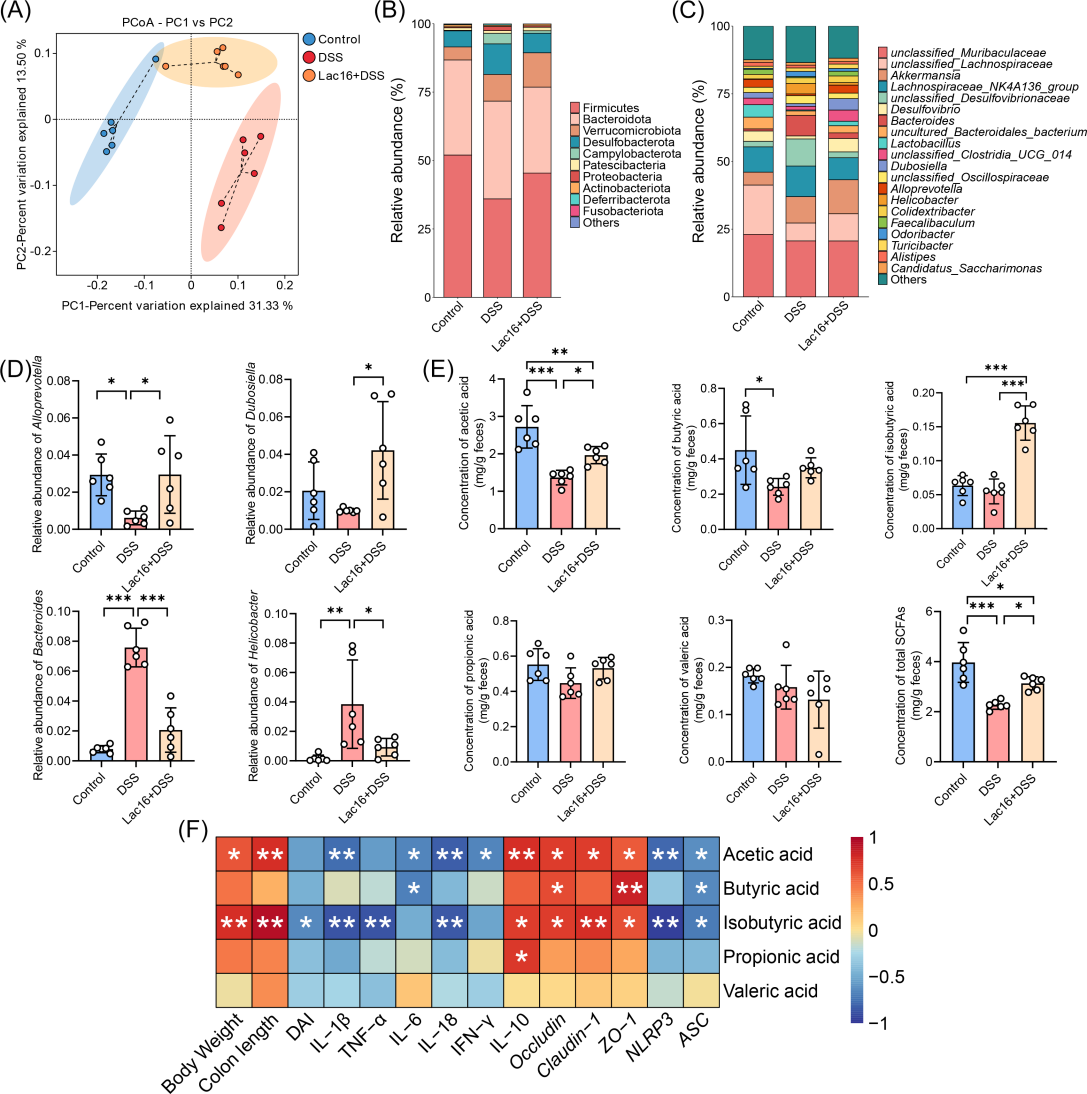
Lac16 pretreatment modified the microbial composition and altered SCFA production of experiment I. (**A**) β-Diversity of microbial communities based on the unweighted_unifrac method. (**B**) Average relative abundance of taxa at the phylum level. (**C**) Average relative abundance of taxa at the genus level. (**D**) Comparison of the relative abundance of *Alloprevotella*, *Dubosiella*, *Bacteroides*, and *Helicobacter*. (**E**) Concentrations of acetic acid, butyric acid, isobutyric acid, propionic acid, valeric acid, and total SCFAs in fecal samples. (**F**) Spearman correlation analysis between the SCFAs and phenotypes. (**A–F**) *n* = 6. Means ± SD; *P < 0.05, **P < 0.01, ***P < 0.001. Significance was determined by one-way analysis of variance with Tukey’s post hoc test.

To investigate the microbiota-dependent mechanisms underlying Lac16-mediated colitis amelioration, we systematically analyzed gut microbial composition in DSS-treated mice with or without Lac16 pretreatment. The current analysis revealed no significant alteration in α-diversity between the Lac16 + DSS and the DSS group (Fig. S3). β-Diversity analysis revealed significant compositional differences among the three groups, as demonstrated by distinct clustering in PCoA plots using the unweighted_unifrac method. Notably, the Lac16 + DSS group showed clear separation from the DSS group, indicating distinct microbial community structures resulting from probiotic intervention (Fig. 6A). Phylum-level analysis demonstrated that DSS treatment significantly decreased the relative abundance of Firmicutes while increasing the abundances of Desulfobacterota, Campylobacterota, and Proteobacteria compared to the control group (Fig. 6B). Compared to the DSS group, the Lac16 + DSS group showed a significantly increased relative abundance of Firmicutes and decreased proportions of Desulfobacterota, Campylobacterota, Proteobacteria, and Deferribacterota. At the genus level, DSS treatment significantly increased the relative abundances of *Bacteroides* and *Helicobacter* while decreasing *Lactobacillus* and *Alloprevotella*. In contrast, Lac16 supplementation in DSS-treated mice substantially enriched *Dubosiella* and *Alloprevotella* and reduced *Bacteroides* and *Helicobacter* compared to DSS controls (Fig. 6C and D).

### Lac16 pretreatment alleviated the reduction of fecal SCFA contents caused by DSS treatment

SCFAs, key microbial metabolites produced by intestinal commensal bacteria, play an essential role in maintaining and restoring intestinal epithelial barrier integrity following mucosal damage (31). Our analysis revealed that DSS treatment significantly decreased concentrations of acetic acid, butyric acid, and total SCFAs. Lac16 pretreatment attenuated these reductions, maintaining higher levels of acetic acid and total SCFAs compared to DSS controls. Notably, isobutyric acid levels were uniquely elevated in the Lac16 + DSS group relative to both control and DSS-treated mice (Fig. 6E). Spearman correlation analysis of combined data from the DSS and Lac16 + DSS groups (Fig. 6F) demonstrated that acetic acid and isobutyric acid levels were positively correlated with body weight, colon length, IL-10 concentration, and expression of tight junction genes (occludin, claudin-1, and zo-1). Conversely, acetic acid and isobutyric acid showed significant negative correlations with pro-inflammatory cytokines (acetic acid: IL-1β, IL-6, IL-18, and IFN-γ; isobutyric acid: IL-1β, TNF-α, and IL-18), disease activity index (isobutyric acid only), and NLRP3 inflammasome components (NLRP3 and ASC gene expression). Butyric acid showed significant positive correlations with occludin and zo-1 mRNA expression while exhibiting strong negative correlations with IL-6 concentration and ASC gene expression. Propionic acid demonstrated a specific positive correlation with IL-10 concentration. Collectively, these findings indicate that Lac16’s therapeutic effects on colitis are mediated through microbiota-derived SCFAs, which collectively modulate epithelial barrier integrity and inflammatory responses.

### Isobutyric acid protected mice against colitis

**Fig 7:**
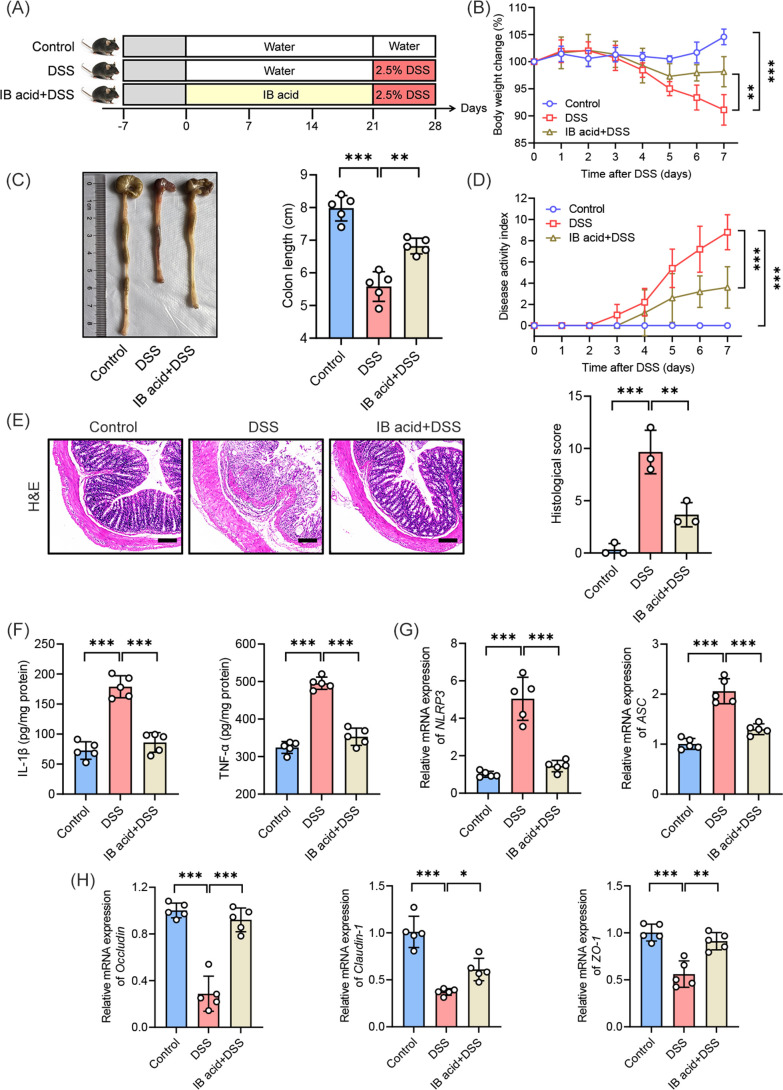
Isobutyric acid protected mice against colitis (**A**) Experimental design of experiment IV (IB acid for isobutyric acid, the same below). (**B**) Body weight change. (**C**) Representative pictures of colons and the lengths of the colons. (**D**) DAI assessment. (**E**) Representative micrographs of H&E (scale bar, 100 µm) and histological score. (**F**) Concentrations of the IL-1β and TNF-α in the colon. (**G**) Relative gene levels of NLRP3 and ASC. (**H**) Relative gene levels of occludin, claudin-1, and zo-1. (**B–H**) *n* = 3 or 5. Means ± SD; *P < 0.05, **P < 0.01, ***P < 0.001. Significance was determined by one-way analysis of variance with Tukey’s post hoc test.

The aforementioned three investigations demonstrated that Lac16-mediated modulation of gut microbiota is essential for colitis remission, with a particularly notable elevation in fecal isobutyric acid levels observed in Lac16-pretreated mice. As a microbiota-derived metabolite, isobutyric acid exhibited distinct correlations with multiple colitis-associated phenotypic and inflammatory cytokines. These results suggest that isobutyric acid may serve as a key mediator of Lac16’s therapeutic effects against colitis. To directly assess isobutyric acid’s anticolitis effects, mice received 150 mM isobutyric acid in drinking water for 3 weeks prior to and during DSS challenge (Fig. 7A). Isobutyric acid pretreatment significantly ameliorated DSS-induced colitis, as evidenced by attenuated body weight loss, preserved colon length, reduced disease activity index scores, and improved histopathological damage compared to the DSS group (Fig. 7B through E). Isobutyric acid pretreatment significantly downregulated pro-inflammatory gene expression (IL-1β, TNF-α, NLRP3, and ASC) while upregulating tight junction components (occludin, claudin-1, and zo-1), demonstrating its protective role against DSS-induced colitis (Fig. 7G and H).

### Isobutyric acid alleviated the inflammation upon LPS challenge *in vitro*

**Fig 8:**
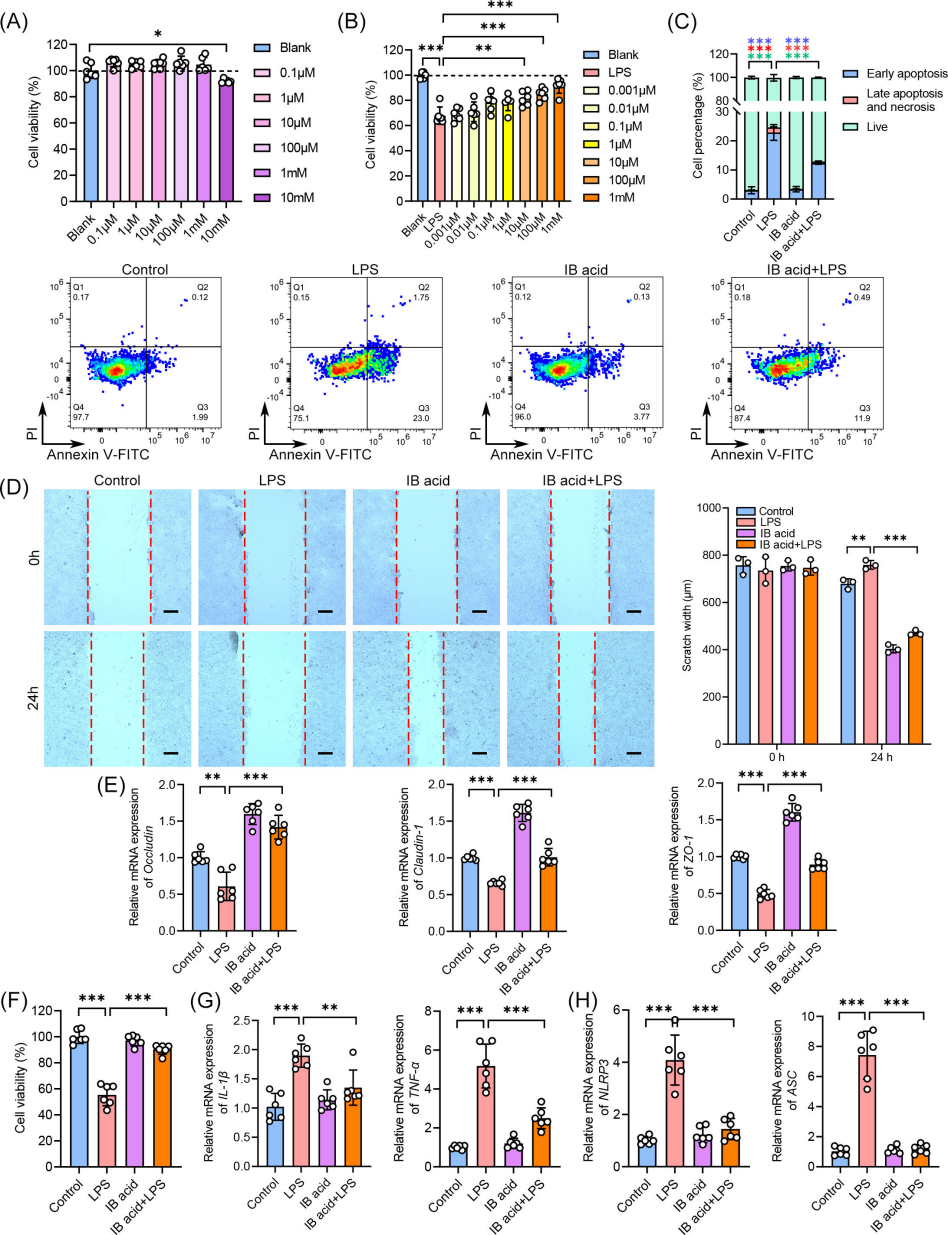
Isobutyric acid intervention alleviated LPS-induced impairment and inflammation *in vitro*. (**A**) Evaluation of cytotoxicity of isobutyric acid in HT-29 cells. (**B**) Evaluation of protective effect of isobutyric acid in HT-29 cells. (**C**) Flow cytometry detection and proportion of Annexin V-FITC/PI labeled HT29 cells. (**D**) Representative micrographs and quantification of scratch wound healing assay in HT-29 cells (scale bar, 200 µm). (**E**) Relative gene levels of occludin, claudin-1, and zo-1 in HT-29 cells. (**F**) Evaluation of protective effect of isobutyric acid in BMDMs. (**G**) Relative gene levels of IL-1β and TNF-α in BMDMs. (**H**). Relative gene levels of NLRP3 and ASC in BMDMs. (F–H) *n* = 3 or 6. Means ± SD; *P < 0.05, **P < 0.01, ***P < 0.001. Significance was determined by one-way analysis of variance with Tukey’s post hoc test.

To investigate isobutyric acid’s direct effects on intestinal epithelial cells and macrophages, we established LPS-stimulated HT-29 cells and BMDM models *in vitro*. Isobutyric acid (≤1 mM) showed no cytotoxicity in HT-29 cells (Fig. 8A) but dose-dependently protected against LPS-induced damage (10 µM–1 mM, Fig. 8B). Isobutyric acid (1 mM) significantly reduced both early apoptosis (Annexin V-FITC^+^/PI^−^) and late apoptosis/necrosis (Annexin V-FITC^+^/PI^+^) in LPS-stimulated HT-29 cells (Fig. 8C). The scratch wound assay demonstrated that isobutyric acid significantly accelerated wound closure in LPS-stimulated HT-29 cells (Fig. 8D). Consistent with *in vivo* observations, isobutyric acid upregulated mRNA expression of tight junction components (occludin, claudin-1, and zo-1) *in vitro* (Fig. 8E). In LPS-activated BMDMs, 1 mM isobutyrate significantly attenuated LPS-induced cytotoxicity while maintaining baseline cell viability (Fig. 8F). Furthermore, it markedly downregulated expression of pro-inflammatory cytokines (TNF-α and IL-6, Fig. 8G) and inflammasome components (NLRP3 and ASC, Fig. 8H). Collectively, these findings further validated that isobutyric acid attenuates inflammatory responses and restores intestinal epithelial homeostasis through its dual actions on immune modulation and barrier reinforcement.

## DISCUSSION

The current investigation demonstrated that Lac16 could alleviate colitis by suppressing NLRP3 inflammasome overactivation via microbiota-derived isobutyric acid. Specifically, Lac16 pretreatment ameliorates colitis through microbiota-dependent mechanisms, including preservation of intestinal barrier integrity, attenuation of inflammatory responses, regulation of macrophage polarization, modulation of NLRP3 inflammasome activation, restoration of microbial homeostasis, and enhancement of beneficial SCFA production. Notably, isobutyric acid—a microbiota-derived metabolite elevated by Lac16 treatment—emerged as a pivotal mediator of the probiotic’s anticolitis effects. Collectively, these findings demonstrated that intestinal microbiota and its metabolites induced by Lac16 played a crucial role in mitigating colitis.

A well-formed intestinal barrier is essential for maintaining intestinal homeostasis (32). Conversely, disturbances in critical barrier components, including tight junction integrity disruption or goblet cell depletion, lead to intestinal dysfunction (33). Furthermore, alterations in the expression and distribution of tight junctions have been regarded as critical factors in colitis pathogenesis (1). In this investigation, Lac16 effectively ameliorated colitis symptoms, including morphological damage, structural abnormalities, goblet cell depletion, and reduced tight junction protein expression.

Restoring the aberrant inflammatory environment represents an effective therapeutic strategy for colitis (34). Lac16 pretreatment significantly decreased immune cell counts in hematological parameters, MPO activity, and pro-inflammatory cytokine levels in colonic tissue. Lac16 pretreatment significantly elevated IL-10 levels in mice, effectively mitigating inflammation and maintaining immune homeostasis. These findings align with existing studies demonstrating probiotic-induced increases in this anti-inflammatory cytokine (1, 22). Macrophages play pivotal roles in both inflammation initiation and resolution and are prominently enriched in colonic tissues of IBD patients (35). Lac16 significantly attenuated colitis-induced M1 macrophage polarization while concurrently promoting M2 macrophage polarization. During immune responses, macrophage polarization is closely associated with NLRP3 inflammasome activation (6, 7). The NLRP3 inflammasome plays a critical role in maintaining intestinal homeostasis, and its appropriate activation is essential for regulating inflammation and promoting tissue repair (10). Dysregulation of NLRP3 is associated with the progression of inflammatory diseases, including IBD (36). In the current research, Lac16 significantly suppressed NLRP3 inflammasome overactivation in colitis. These results demonstrate that probiotic Lac16 can mitigate excessive inflammatory responses and associated tissue injury.

The gut microbiota plays a significant role in IBD pathogenesis, with its impact on host immune responses attracting growing research interest (22, 37). Moreover, intestinal microorganisms and their metabolites could affect intestinal mucosal integrity and modulate immune responses (15, 38). In pseudo-germ-free mice with antibiotic-induced microbiota depletion, Lac16 showed impaired ability to suppress NLRP3 inflammasome overactivation and ameliorate colitis. These findings suggest that the intestinal microbiota is essential for Lac16-mediated alleviation of colitis, indicating that the probiotic Lac16 ameliorates colitis symptoms through microbiota modulation. Subsequently, the FMT mouse model was employed to validate the anticolitis effects mediated by Lac16-regulated gut microbiota. Results demonstrated significant amelioration of colitis symptoms in both FMT-Lac16 and FMT-Lac16 + DSS groups. These findings demonstrate that Lac16’s therapeutic effects can be transferred via FMT. Collectively, our results support the conclusion that Lac16 suppresses NLRP3 inflammasome overactivation and alleviates colitis in a gut microbiota-dependent manner.

Intestinal inflammation induces alterations in the intestinal environment and the microbial composition. Meanwhile, these gut microbiota alterations reciprocally modulate host immune responses (39). The current investigation revealed significant alterations in the composition and organization of the microbiota, as shown by the β-diversity of bacteria, between the Lac16 + DSS and DSS groups. At the phylum level, the observed reduction in Firmicutes abundance, coupled with increased Proteobacteria prevalence, was strongly correlated with colitis pathogenesis and progression (40). Notably, Proteobacteria represents a well-established microbial marker of gut dysbiosis (41), whereas Desulfobacterota promotes pro-inflammatory cytokine production and aggravates colitis pathogenesis (42). Lac16 pretreatment significantly attenuated the dysbiosis-associated shifts in phylum-level abundance. At the genus level, Lac16 treatment significantly reduced the colitis-associated expansion of *Bacteroides* and *Helicobacter* populations. While *Bacteroides* represents an important commensal bacterium implicated in IBD pathogenesis (43), *Helicobacter* has similarly been linked to IBD development (44). Concurrently, Lac16 treatment markedly elevated *Dubosiella* abundance while preventing the depletion of *Alloprevotella*, two SCFA-producing genera whose relative abundance inversely correlates with intestinal inflammation (45, 46). Surprisingly, Lac16 treatment did not alter *Lactobacillus* abundance in colitic mice, suggesting the need for third-generation sequencing in our future studies to resolve bacterial compositional changes at species-level resolution. Collectively, these results demonstrate that Lac16 pretreatment effectively ameliorates colitis-induced microbiota dysbiosis and associated pathological damage.

Microbiota-derived SCFAs, highly abundant in the intestinal lumen, play crucial roles in maintaining intestinal barrier integrity and function, regulating immune cell activity, and promoting mucosal homeostasis (47). Multiple studies showed that UC patients harbor significantly lower abundances of SCFA-producing bacteria in both mucosal and fecal samples, along with reduced SCFA concentrations, relative to healthy individuals (48, 49). In this study, Lac16 treatment significantly restored colitis-induced reductions in acetic acid and total SCFA levels while markedly elevating isobutyric acid concentration. Correlation analyses revealed that SCFA levels—particularly acetic acid, butyric acid, and isobutyric acid—showed significant positive associations with colitis-alleviating phenotypes and negative associations with colitis-promoting phenotypes. Given the well-documented protective effects of microbiota-derived acetic acid and butyric acid against colitis (50–57), we focused subsequent investigations on isobutyric acid, a microbial fermentation product (2-methylpropanoic acid) belonging to the SCFA family, for its potential anticolitis properties (58). Emerging evidence has identified elevated isobutyric acid levels as correlating with colitis remission, positioning this microbial metabolite as a potential therapeutic response biomarker (59). On this basis, our experimental results demonstrated that isobutyric acid pretreatment effectively suppressed NLRP3 inflammasome overactivation and protected against colitis in murine models, mirroring the therapeutic effects observed with Lac16 administration as indicated by significant amelioration of clinical symptoms. Consistent with these observations, *in vitro* experiments demonstrated that isobutyric acid attenuated LPS-induced cellular injury and inflammatory responses, enhanced wound healing capacity, upregulated tight junction gene expression, reduced pro-inflammatory cytokine production, and suppressed NLRP3 inflammasome overactivation. Collectively, these findings demonstrate that isobutyric acid, a microbiota-derived SCFA, plays a pivotal role in suppressing NLRP3 inflammasome overactivation and mediates the protective effects of Lac16 against colitis pathogenesis. Nevertheless, the precise immunomodulatory mechanisms through which Lac16-derived microbiota metabolites, particularly isobutyric acid, confer colitis protection require further elucidation. Further comprehensive research is required to perform reverse validation using bacterial strains with deficient isobutyric acid synthesis pathways and to investigate the synergistic interactions among gut microbiota-derived metabolites. Additionally, rigorous trials evaluating Lac16 administration after colitis induction are needed to comprehensively assess its therapeutic potential.

### Conclusion

**Fig 9:**
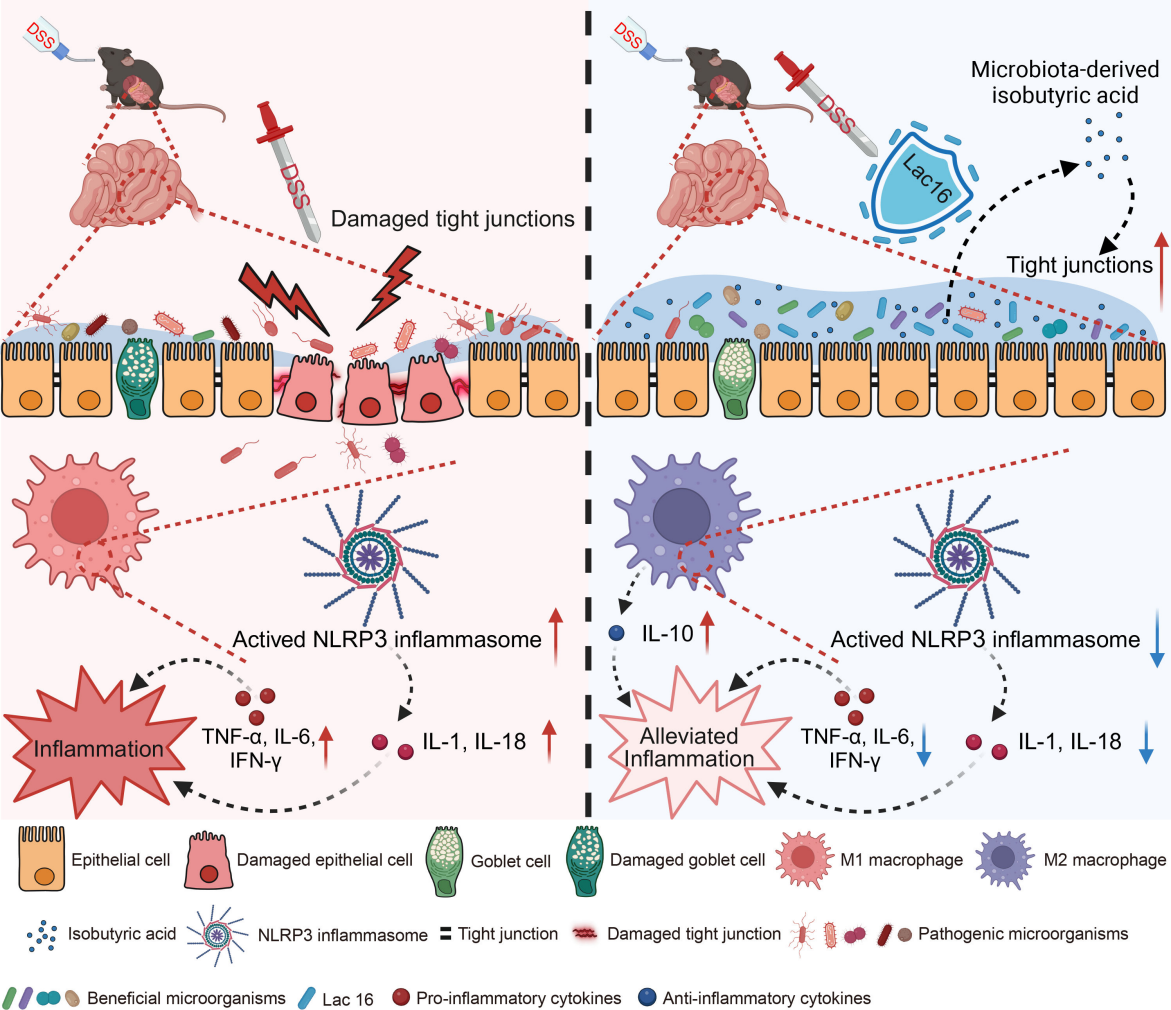
The schematic diagram of the process by which Lac16 alleviated DSS-induced colitis.

In summary, our findings demonstrate that Lac16 alleviates DSS-induced colitis by preserving intestinal barrier integrity, reducing inflammation, regulating macrophage polarization, modulating NLRP3 inflammasome activation, and maintaining gut microbiota homeostasis. Crucially, Lac16’s suppression of NLRP3 inflammasome overactivation depends on gut microbiota, with isobutyric acid serving as a key mediator of this protective effect (Fig. 9). This study elucidates novel mechanisms underlying Lac16’s dual modulation of host immunity and gut microbiota, positioning it as a potential therapeutic strategy for IBD management.

## Data Availability

All data generated or analyzed during this study are available from the corresponding author upon request. Raw sequence data of microbiota which support the findings in our study have been deposited into National Center for Biotechnology Information’s Sequence Read Archive under accession number PRJNA1332556.

## SUPPLEMENTAL MATERIAL

Supplemental material mbio.02392-25-s0001.docx Supplemental figures and table.

ASM does not own the copyrights to Supplemental Material that may be linked to, or accessed through, an article. The authors have granted ASM a non-exclusive, world-wide license to publish the Supplemental Material files. Please contact the corresponding author directly for reuse.
