# Supplementary material for: Lactobacillus plantarum Lac16 alleviates dextran sodium sulfate-induced colitis in mice by suppressing NLRP3 inflammasome overactivation through microbiota-derived isobutyric acid
Source: mBio. 2025 Oct 31;16(12):e02392-25. doi: 10.1128/mbio.02392-25 (PMC12691655; doi:10.1128/mbio.02392-25)
Supplement: Supplemental material — Supplemental figures and table. [file mbio.02392-25-s0001.docx]

Figure S1


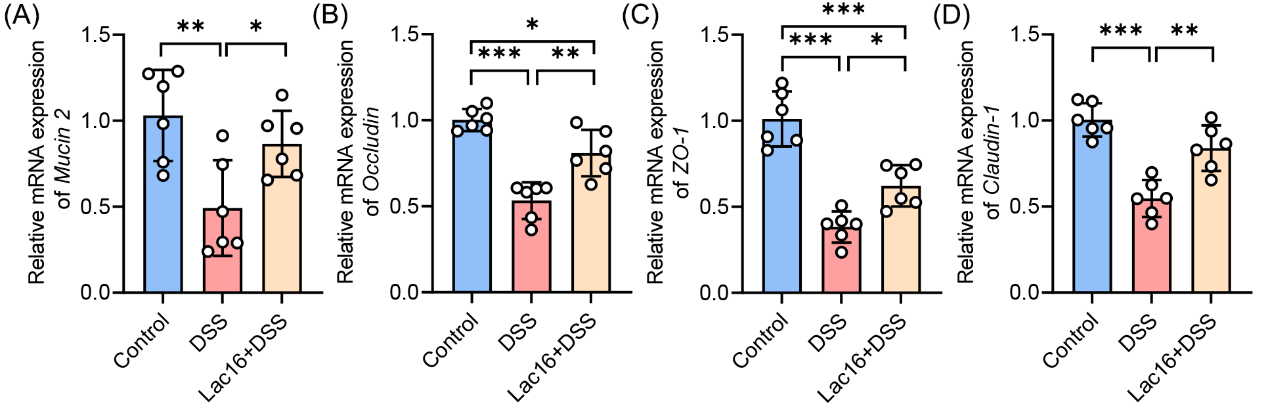


Figure S1. Relative gene expression levels of Mucin 2 (A), Occludin (B), ZO-1 (C), and Claudin-1 (D) in Experiment Ⅰ. (A to D) *n* = 6 per group. Data are presented as the means ± SD; * *p* < 0.05, ** *p* < 0.01, *** *p* < 0.001. Significance was determined by one-way ANOVA with Tukey's post-hoc test.

Figure S2


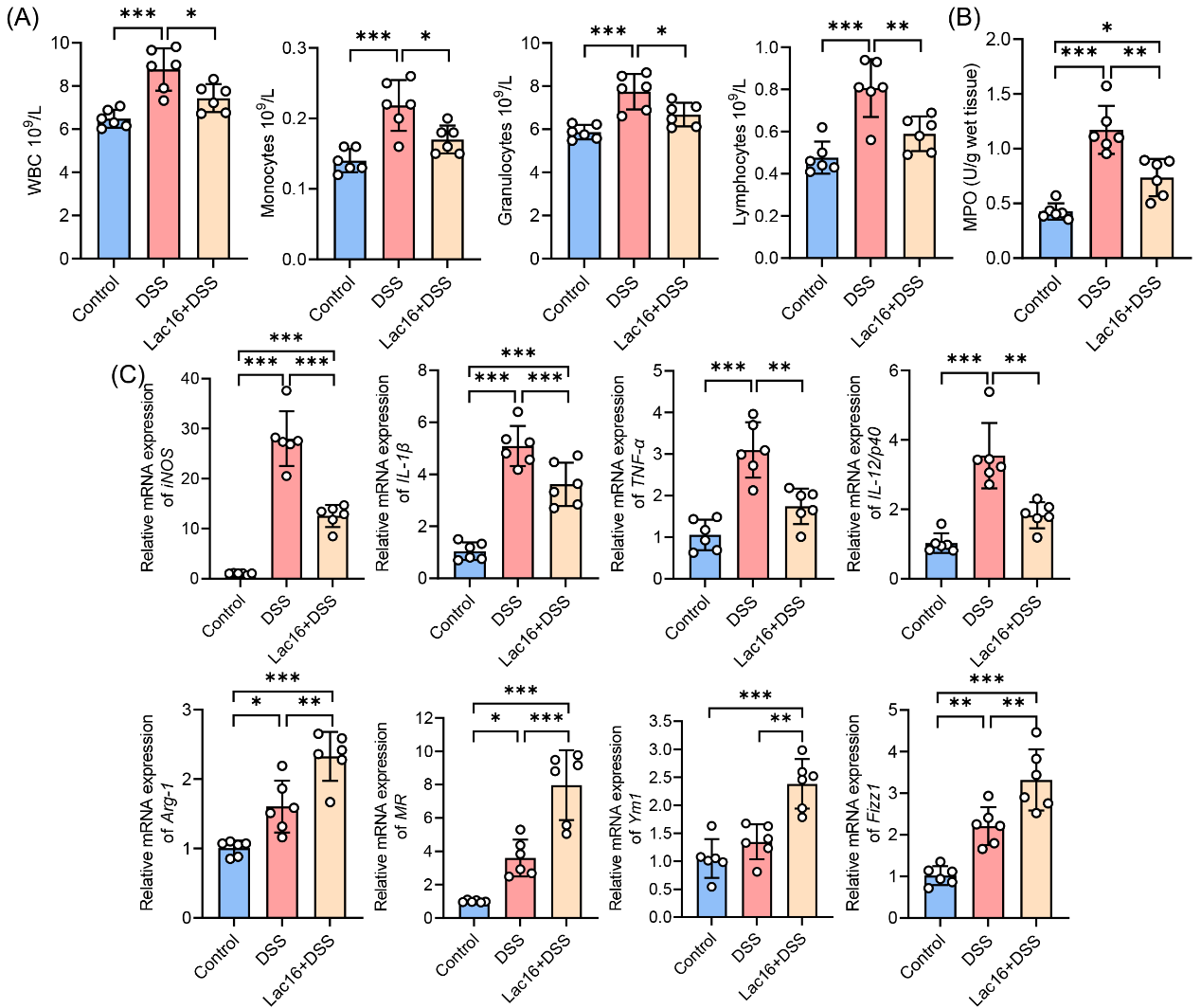


Figure S2. (A) The number of white blood cells, monocytes, granulocytes, and lymphocytes in the blood in Experiment Ⅰ. (B) MPO activity of colon tissue in Experiment Ⅰ. (C) Relative gene expression levels of iNOS, IL-1β, TNF-α, IL-12/p40, Arg-1, MR, Ym1, and Fizz1 in the colon in Experiment Ⅰ. (A to C) *n* = 6 per group. Data are presented as the means ± SD; * *p* < 0.05, ** *p* < 0.01, *** *p* < 0.001. Significance was determined by one-way ANOVA with Tukey's post-hoc test.

Figure S3


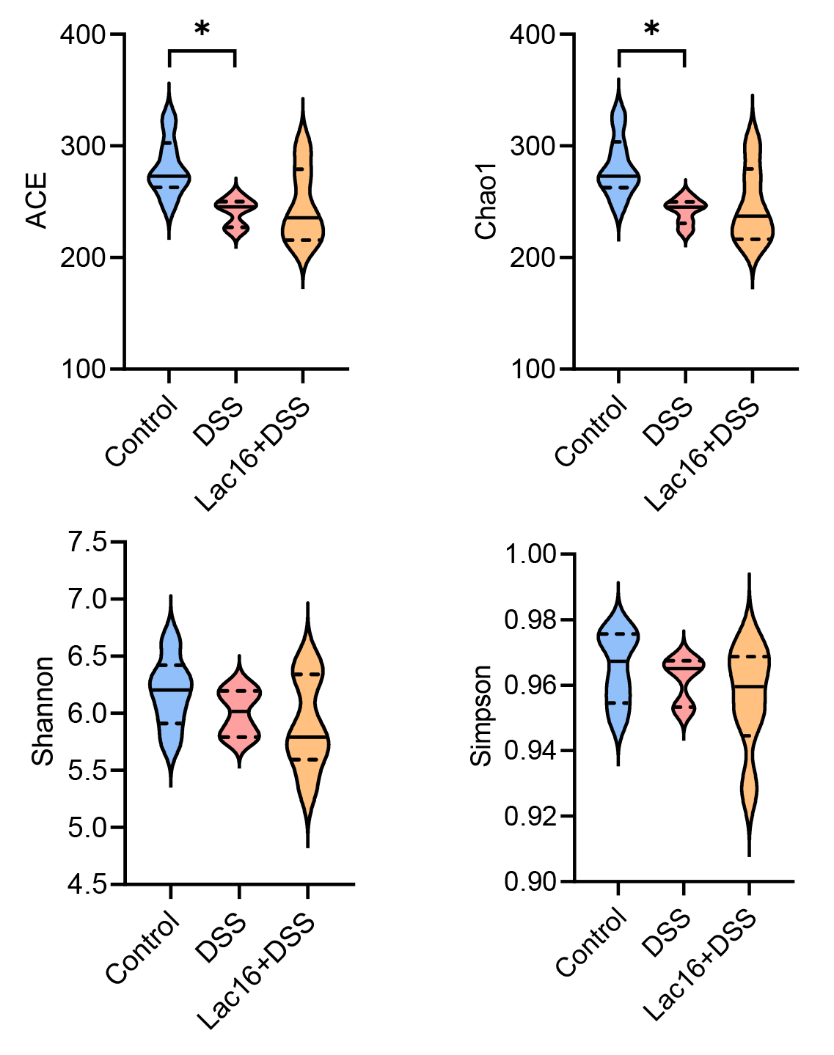


Figure S3. *α*-diversity of bacterial communities represented by Ace, Chao1, Shannon, and Simpson in Experiment Ⅰ. *n* = 6 per group. Data are presented as the means ± SD; * *p* < 0.05, ** *p* < 0.01, *** *p* < 0.001. Significance was determined by one-way ANOVA with Tukey's post-hoc test.

Table S1. Primers used for quantitative real-time PCR.

| Gene Name | Forward Sequence  (5′→3′) | Reverse Sequence  (5′→3′) |
| --- | --- | --- |
| m-β-actin | GCTTCTAGGCGGACTGTTACT | GCCTTCACCGTTCCAGTTTTT |
| m-Mucin 2 | GCCCACCTCACAAGCAGTAT | GTCATAGCCAGGGGCAAACT |
| m-Occludin | TTCAAAAGGCCTCACGGACA | TTGAACTGTGGATTGGCAGC |
| m-ZO-1 | AGAAATCCTTTCACACCTACTGA | GCACATCAGCACGATTTCTGT |
| m-Claudin-1 | TCATGCCAATGGTGGACACA | GACTGTGGATGTCCTGCGTT |
| m-iNOS | CTCACCTACTTCCTGGACATTAC | CAATCTCTGCCTATCCGTCTC |
| m-IL-1β | TGCCACCTTTTGACAGTGATG | ATGTGCTGCTGCGAGATTTG |
| m-TNF-α | CCTCACACTCACAAACCACCA | ACAAGGTACAACCCATCGGC |
| m-IL12/p40 | CCCATTCCTACTTCTCCCTCAA | CCTCCTCTGTCTCCTTCATCTT |
| m-Arg1 | GCCTTTGTTGATGTCCCTAATGA | CCACACTGACTCTTCCATTCTTC |
| m-MR | TTCAGCTATTGGACGCGAGG | GAATCTGACACCCAGCGGAA |
| m-Ym1 | ACTCCTCAGAACCGTCAGAT | GTAGCAGCCTTGGAATGTCTTT |
| m-Fizz1 | TCGTGGAGAATAAGGTCAAGGAA | CGAGTAAGCACAGGCAGTTG |
| m-NLRP3 | GTACCCAAGGCTGCTATCTGG | GGACACTCGTCATCTTCAGCA |
| m-ASC | GACAGTACCAGGCAGTTCGT | AGTCCTTGCAGGTCAGGTTC |
| h-β-actin | CCTCGCCTTTGCCGATCC | CGCGGCGATATCATCATCC |
| h-IL-1β | AGCTACGAATCTCCGACCAC | CGTTATCCCATGTGTCGAAGAA |
| h-TNF-α | CAAGGACAGCAGAGGACCAG | TGGCGTCTGAGGGTTGTTTT |
| h-Occludin | AGTGCCACTTTGGCATTATGAG | CTTGTGGCAGCAATTGGAAAC |
| h-Claudin-1 | GGGCAGATCCAGTGCAAAG | GGATGCCAACCACCATCAAG |
| h-ZO-1 | GACCAATAGCTGATGTTGCCAG | TGCAGGCGAATAATGCCAGA |

Notes: m for mice; h for human.
